# Supplementary material for: Néel-type skyrmion in WTe2/Fe3GeTe2 van der Waals heterostructure
Source: Nat Commun. 2020 Jul 31;11:3860. doi: 10.1038/s41467-020-17566-x (PMC7395126; doi:10.1038/s41467-020-17566-x)
Supplement: Supplementary file 1 — Supplementary Information [file 41467_2020_17566_MOESM1_ESM.pdf]

Supplementary Information
Néel-type Skyrmion in WTe_2/Fe_3GeTe_2 van der Waals heterostructure

Yingying Wu et al.

Supplementary Note 1. Hall resistivity of monolayer WTe_2

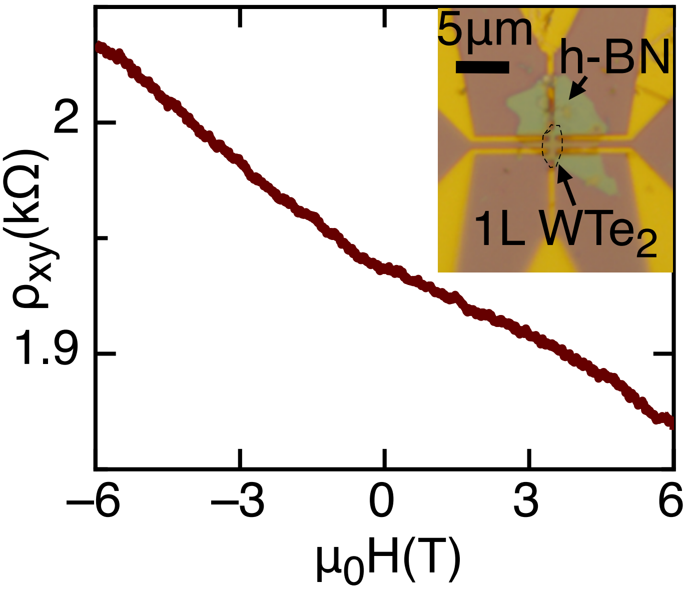

Supplementary Figure 1: Hall resistivity of a monolayer WTe_2 on bottom electrodes at 2 K.

To confirm the dip and peak near the magnetic transition edge in the transport signal as shown in Fig. 2 in the main text are from the topological Hall effect, we have shown the Hall resistivity of h-BN/monolayer WTe_2 in Supplementary Fig. 1. This helps exclude the possibility that the dip and peak are from the transport signal of WTe_2 and thus leads to the conclusion that topological Hall effect exists at the interface.

Supplementary Note 2. Device fabrication

We have prepared the bottom electrodes by e-beam lithography first. Then 5/30 nm Cr/Au was evaporated to form bottom electrodes. Then we exfoliated WTe_2 and FGT from high-quality bulk materials separately onto the 300 nm SiO_2/Si substrates. After that, PDMS/PPC on a glass slide was used to pick up the monolayer or bilayer WTe_2 on the substrate. The pick-up procedure was to heat the sample stage up to 50°C when PDMS/PPC was lowered to touch WTe_2 and shut down the heating while detaching the PDMS/PPC from the sample stage. After the separation, the WTe_2 was picked up by PDMS/PPC. Then PDMS/PPC/WTe_2 was used to pick up FGT thin layers. The resulted

PDMS/PPC/WTe_2/FGT was then transferred onto the prepared bottom metal electrodes with proper alignment. After removing the PDMS/PPC by acetone, the WTe_2/FGT heterostructures are in good contact with the bottom electrodes. Finally, we always transferred h-BN thin layers onto this structure to protect it from effects of the ambient conditions. All the procedures were carried out inside a glove box, with H_2O of 1.2 ppm (parts per million) and O_2 less than 50 ppm.

Supplementary Note 3. ρ-T curves for FGT with varied thickness

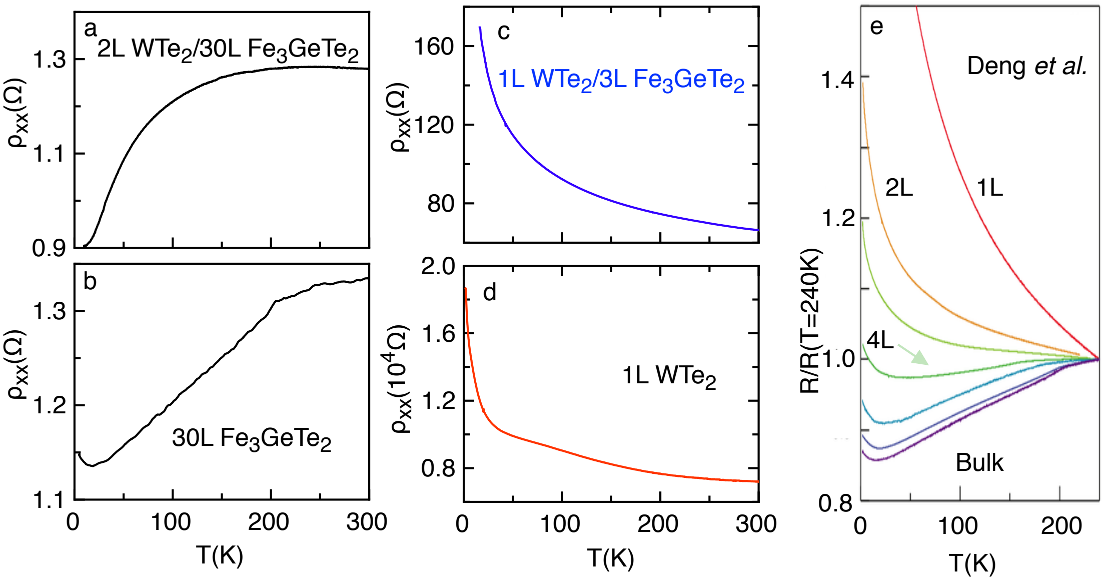

Supplementary Figure 2: Temperature (T) dependence of longitudinal resistivity (ρ_{xx}). a, The $\rho_{xx} - T$ curve for a 30L Fe_3GeTe_2 film with a 2L WTe_2 capping. b, The $\rho_{xx} - T$ result of the control sample, where a bare 30L Fe_3GeTe_2 film is not capped with WTe_2. c, The $\rho_{xx} - T$ behavior of a 3L Fe_3GeTe_2 thin film with a 1L capping WTe_2. d, The $\rho_{xx} - T$ for a control sample with a 1L WTe_2 film. e, Previous $R_{xx} - T$ measurements of Fe_3GeTe_2 films on Al_2O_3 thin films done in Ref. [1].

As the thickness decreases, the resistivity dependence on the temperature of FGT films changes from metallic to insulating with an Al_2O_3-assisted exfoliation method, as has been shown before[1]. To confirm this transition, we have fabricated additional samples and carried out similar measurements in our WTe_2/FGT heterostructures. Here, Supplementary

Fig. 2a demonstrates the $\rho_{xx} - T$ behavior for a 30L FGT capped with a 2L WTe_2. Such metallic $\rho_{xx} - T$ behavior is mainly contributed by the 30L FGT layer, as shown by the control sample without the WTe_2 capping (Supplementary Fig. 2b).

As the thickness of FGT goes down to 3L, the resistivity increases when temperature decreases, as shown in Supplementary Fig. 2c. As a comparison, we obtained the $\rho_{xx} - T$ measurement for a monolayer WTe_2, which is roughly two orders of magnitude more insulating than the heterostructure. We therefore conclude that the carrier transport in Supplementary Fig. 2c is also dominated by the FGT layer, which is not only ∼2 orders of magnitude more insulating than the case of a 30L FGT, but also presents a semiconducting $\rho_{xx} - T$ trend. These observations are consistent with existing results, where uncapped FGT films were measured on Al_2O_3, as shown in Supplementary Fig. 2e.

Supplementary Note 4. Antisymmetric ρ_{xx}-B

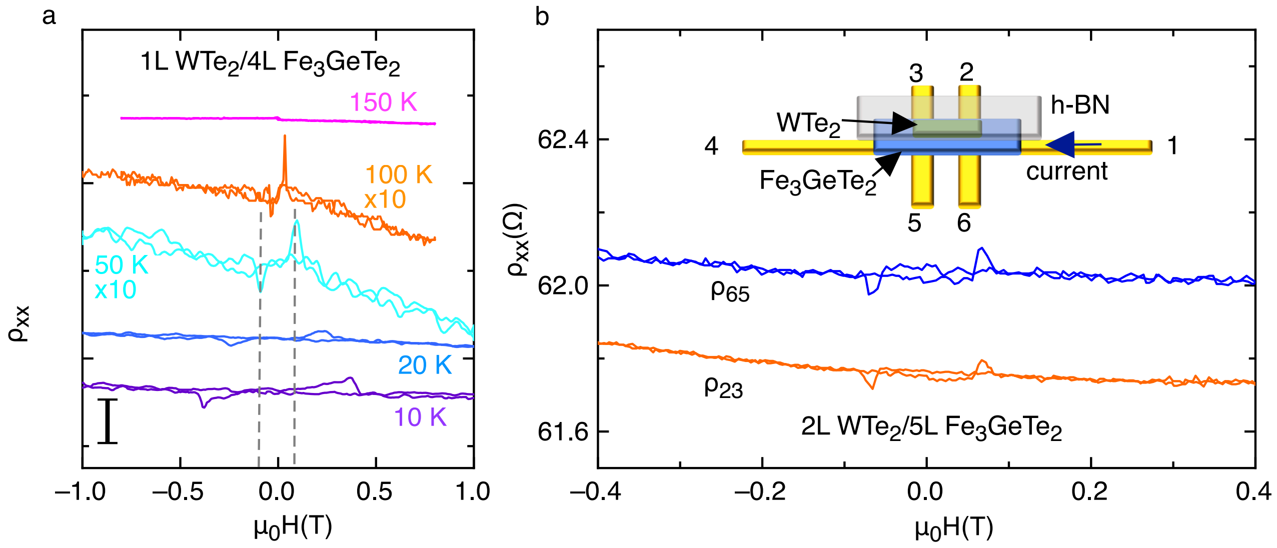

Supplementary Figure 3: Antisymmetric ρ_{xx}-B in WTe_2/Fe_3GeTe_2. a, Magnetoresistivity for 1L WTe_2/4L Fe_3GeTe_2 heterostructure. Vertical scale bar: 1 Ω. b, Configuration of two ρ_{xx} measured show the same polarity at 100 K.

For the 1L WTe_2/4L FGT heterostructure, the topological Hall signal from transverse resistivity has been shown in Fig. 2c in the main text. However, we also observed an antisymmetric magnetoresistivity (MR) as shown in Supplementary Fig. 3a. Indeed, the

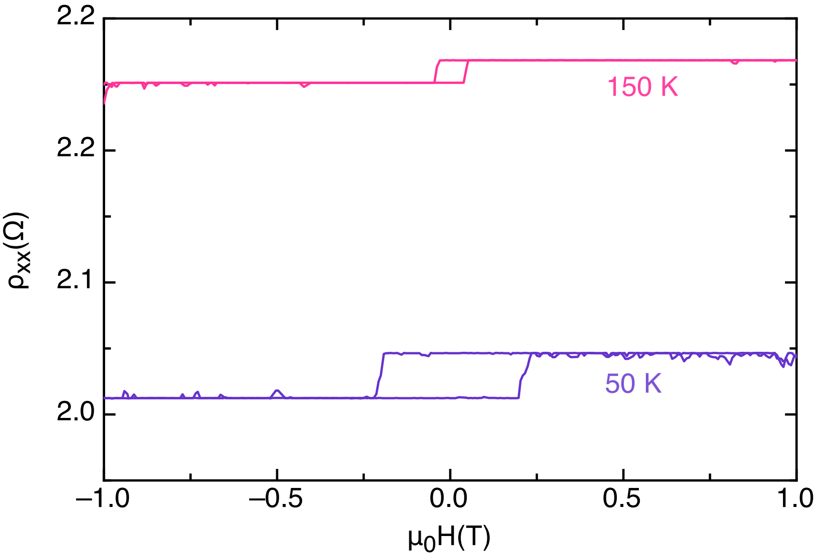

Supplementary Figure 4: Magnetoresistivity shows similar square loop as Hall resistivity (ρ_{xy}) in 60L Fe_3GeTe_2 only.

antisymmetric MR behavior during the magnetic reversal could originate from the electron scattering due to magnetic domain walls in a thin-film magnet with perpendicular anisotropy[2]. If this were true, any misalignment of the transverse electrodes would capture such antisymmetric signal in ρ_{xy} even without skyrmions. To rule out such alternative interpretation, we performed additional measurements, as discussed in the following.

If the antisymmetric behavior in ρ_{xx} came from a domain wall, switching measuring electrodes from ρ_{23} to ρ_{65}, the antisymmetric behavior would have changed polarity[2]. However, this was not the case. The antisymmetric behavior maintained its polarity, namely, a dip on the left and a spike on the right in both cases, as shown in Supplementary Fig. 3b. This rules out the domain wall interpretation.

Indeed, the asymmetric ρ_{xx} captured in our measurement is likely due to the mixing between ρ_{xy} and ρ_{xx}. Such mixing occurs in many 2D material studies since it is difficult to control the geometry of exfoliated van der Waals materials. As shown in Supplementary Fig. 4, the magnetoresistivity of a 60L FGT (on a SiO_2/Si substrate) also possesses hysteresis of $\sim$0.05 Ω. Since it is technically difficult to separate longitudinal and transverse components, we show the unsymmetrized raw data throughout the manuscript.

Supplementary Note 5. Focus change during the L-TEM measurements

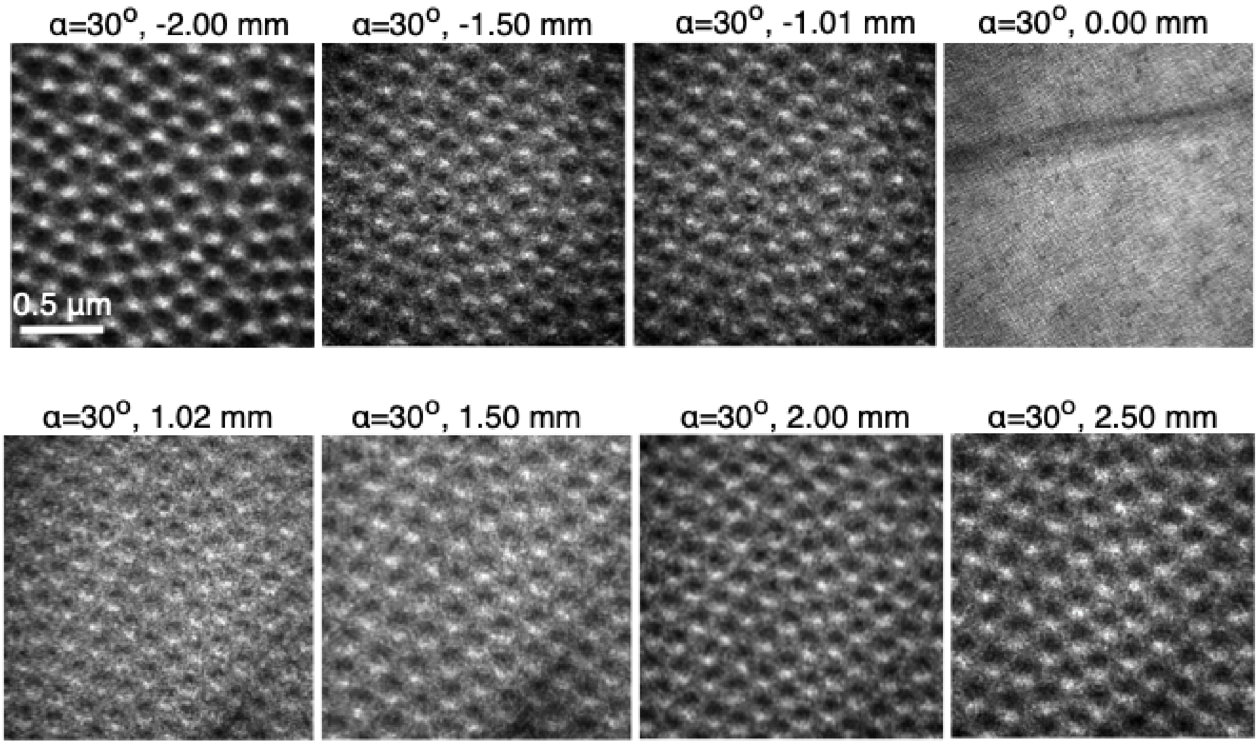

Supplementary Figure 5: Focus was changed from under foucs to over focus on WTe_2/40L Fe_3GeTe_2 samples with a field of 510 Oe at 180 K.

We checked the skyrmion lattice from under focus to over focus for WTe_2/40L FGT at 180 K. Skyrmions are only observed at de-focused images. As shown in Supplementary Fig. 5, the under and over focused L-TEM images exhibit the opposite dark-bright color contrast.

Supplementary Note 6. Skyrmion size

As shown in Supplementary Fig. 6, a line profile is used to analyze the contrast for a skyrmion. The distance between the lowest and highest data points is the skyrmions size.

Supplementary Note 7. DMI at the interface of WTe_2 and FGT

We assume the DMI is mainly enhanced at the interface between WTe_2 and FGT. This is supported by our L-TEM data shown in Supplementary Fig. 7. When the FGT layer is

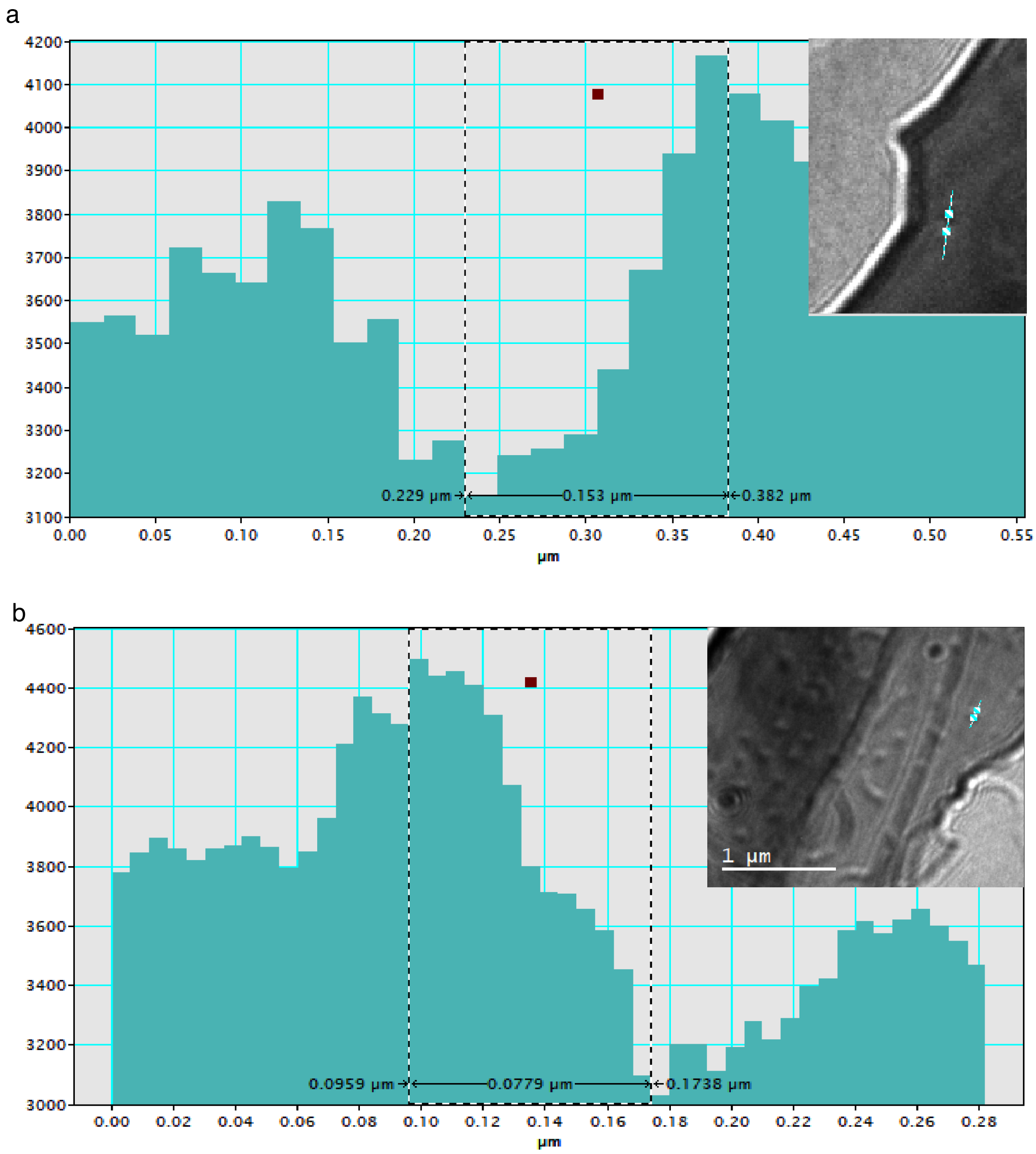

Supplementary Figure 6: Line profile for the image of skyrmions observed in 2L WTe_2/30L Fe_3GeTe_2 samples. a, The skyrmion size is determined to be $\sim$ 150 nm at 94 K with magnetic fields of 540 Oe and 600 Oe. b, The skyrmion size is determined to be $\sim$ 80 nm at 198 K with a magnetic field of 390 Oe.

35 L thick, the stripe domain period is smaller compared to the regions without the WTe_2 capping, as shown in Supplementary Fig. 7a. However, when the FGT layer is $\sim$ 65 L thick, there was no observable difference in the domain width, as shown in Supplementary Fig. 7b. Besides, when the thickness of FGT is reduced to 30L, the interface plays a more

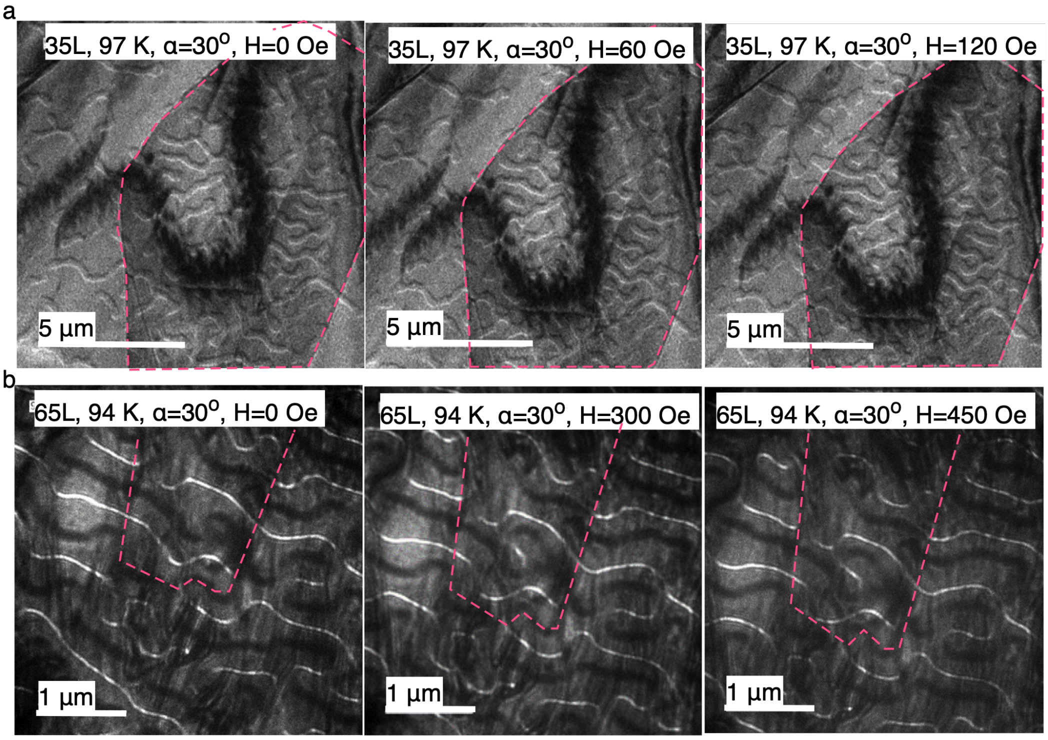

Supplementary Figure 7: Magnetic domain difference between Fe_3GeTe_2 and WTe_2/Fe_3GeTe_2 samples. a, For 35L Fe_3GeTe_2, the region with WTe_2 shows narrower domain widths. b, For 65L Fe_3GeTe_2, there is no magnetic domain difference. The region with dashed line is for Fe_3GeTe_2 with WTe_2.

important role, resulting in denser stripe domains in the WTe_2 capped regions, as shown in Fig. 4 in the main text. This indicates the DMI is more pronounced in thinner FGT with WTe_2. Thus the DMI from the interface can penetrate a finite depth into FGT.

Supplementary Note 8. Measurement of domain width

Bodenberger and Hubert[3] used a stereological method to define the surface magnetic domain width w of complicated or arbitrary magnetic structure patterns. In their method, w is defined as:

$$w = \frac{2 \times total\ \ test\ \ line\ \ length}{\pi \times number\, of\, intersections}, \tag{1}$$

which appears to be the most universal and commonly applied method[4–6]. In this method, an effective domain width is defined as the ratio of total test line length to the number of intersections of domain walls. For the purpose of evaluating the total domain width, four

test straight lines running in random directions is used; the method is illustrated in the image of Supplementary Fig. 8, where four test lines are drawn. The determined domain width is 290 ± 10 nm.

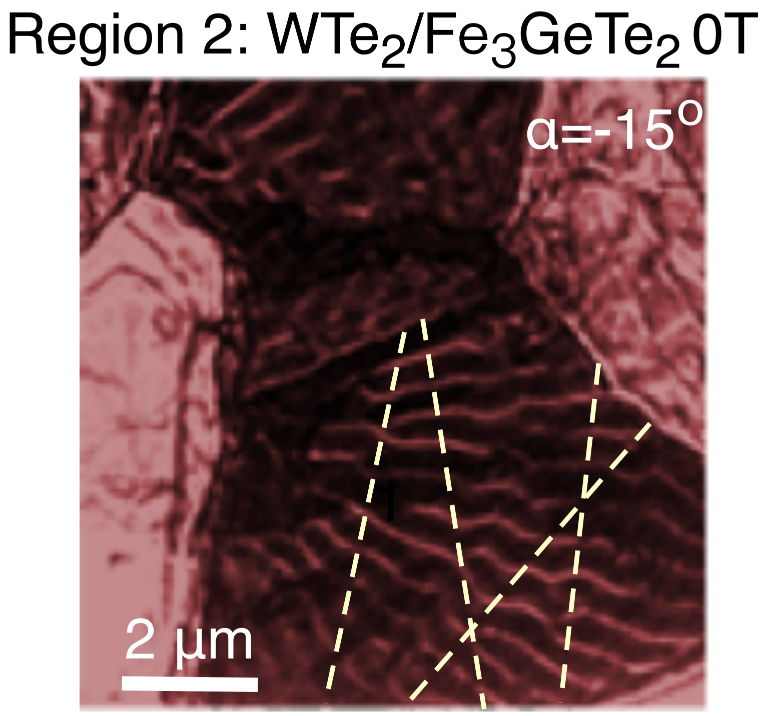

Supplementary Figure 8: Representative image used to obtain the average domain size of WTe_2/30L Fe_3GeTe_2 sample.

Supplementary Note 9. Estimation of DMI constant

Based on the Stoner-Wohlfarth model[7],the uniaxial anisotropy constant K_u can be derived via:

$$\frac{2K_\mathrm{u}}{M_\mathrm{s}} = \mu_0 H_\mathrm{sat}. \tag{2}$$

As shown in Supplementary Fig. 9 for a 2L WTe_2/30L FGT heterostructure, H_sat decreases as the temperature increases. Thus we can determine the ratio of the uniaxial anisotropy constant at 5 K $K_\text{u-5K}$ and at 94 K $K_\text{u-94K}$. Meanwhile, Ref[8] gives the parameters for bulk FGT around 5 K: $M_\text{s-5K}$=376 emu · cm^{-3}, $K_\text{u-5K}$=1.46 × 10^7 erg·cm^{-3}, $A = 10^{-7}$erg·cm^{-1}. Thus $K_\text{u-94K} \sim 9.7 \times 10^6$ erg·cm^{-3} was estimated from Equation 2. Since $K_\mathrm{d} \ll K_\mathrm{u}$, the effective anisotropy constant $K_\text{eff-94K} \sim K_\text{u-94K} \sim 9.7 \times 10^6$ erg·cm^{-3}. As a result, the domain

wall energy for FGT without the DMI contribution is $\sim$3.9 mJm^{-2}. A DMI constant of 1.0 mJm^{-2} is obtained.

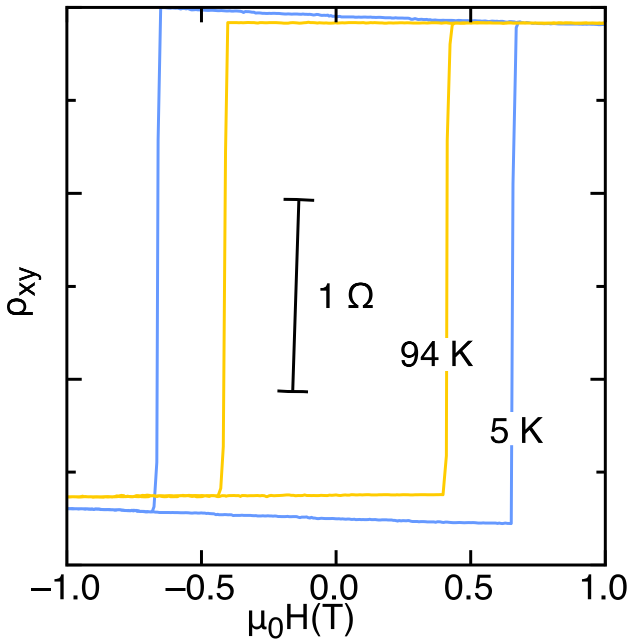

Supplementary Figure 9: Hall resistivity of 2L WTe_2/30L Fe_3GeTe_2 heterostructure at 5 K and 94 K.

Supplementary Note 10. Field dependent magnetic domains for FGT and WTe_2/FGT samples

Here we show how the magnetic domains in WTe_2/FGT differ from FGT in the Supplementary Fig. 10. For the FGT without WTe_2, the magnetization saturates and it enters the ferromagnetic phase when the field is 660 Oe at 195 K, on the other hand, for FGT with WTe_2 a group of skyrmions shows up. For FGT with WTe_2, the DMI penetrates to a depth from the interface and disappears away from the interface in FGT. For the FGT away from the interface, it enters uniform ferromagnetic phase and contributes no contrast. Thus the image captured for WTe_2/FGT is with the skyrmions at the interface.

Besides, Supplementary Fig. 11 shows the magnetic domain evolution for 35L FGT with and without 8L WTe_2 in more details when the magnetic field is varied in the range of 480

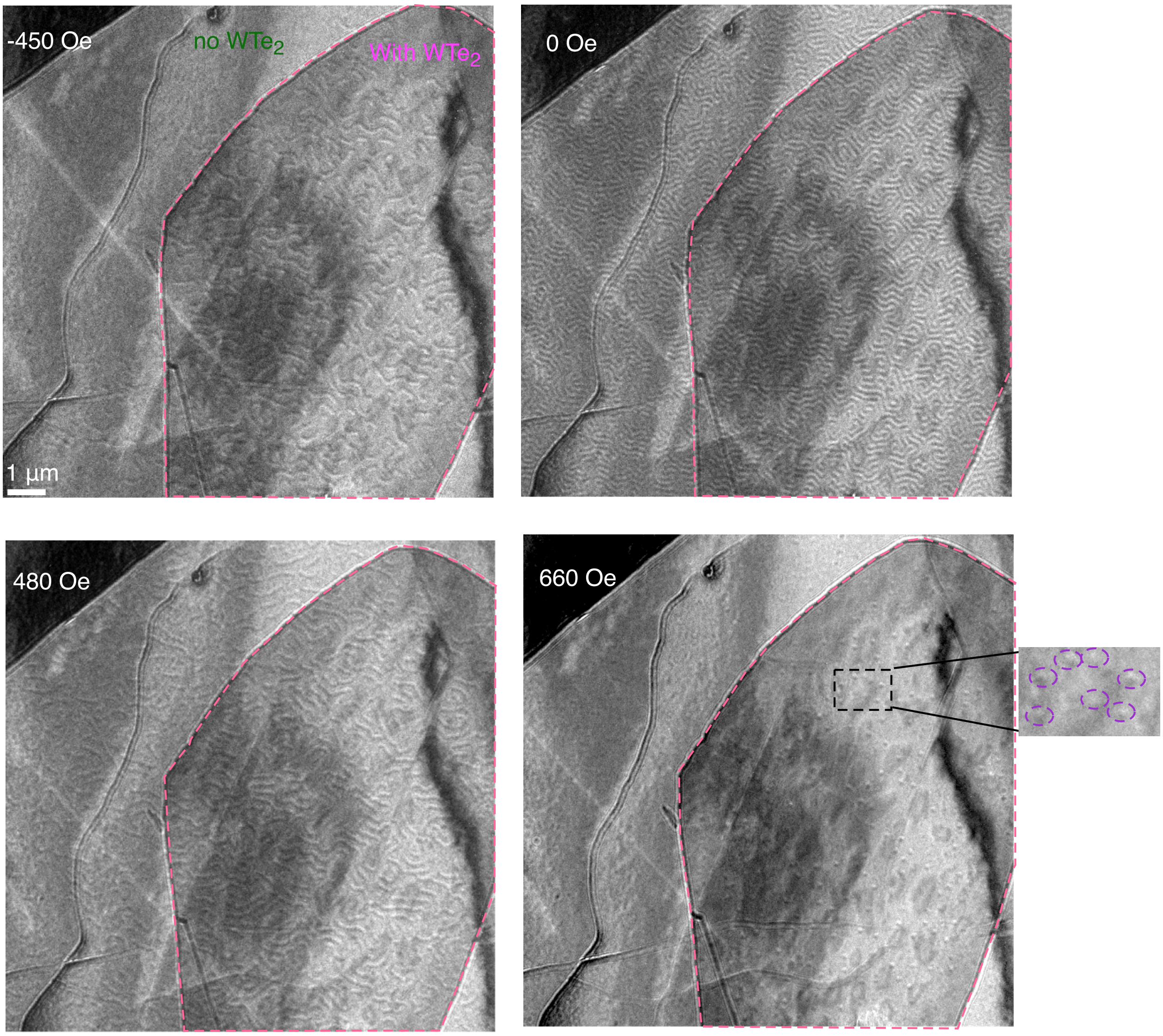

Supplementary Figure 10: Magnetic domain difference for 35L Fe_3GeTe_2 with and without 8L WTe_2 at 195 K with a tilting angle $\alpha = 30^o$ and a varied field. The dashed pink line region is Fe_3GeTe_2 with WTe_2. We have zoomed in and indicated the skyrmions with purple dashed circles.

Oe to 630 Oe. When the magnetic field is increased with a smaller step, the FGT without WTe_2 does not show clear sign of skyrmions and it enters into a uniform single domain directly when the magnetic field is increased. However, skyrmions gradually develop and appear in the FGT with WTe_2 regions with the increasing magnetic field, which is consistent with Supplementary Fig. 10.

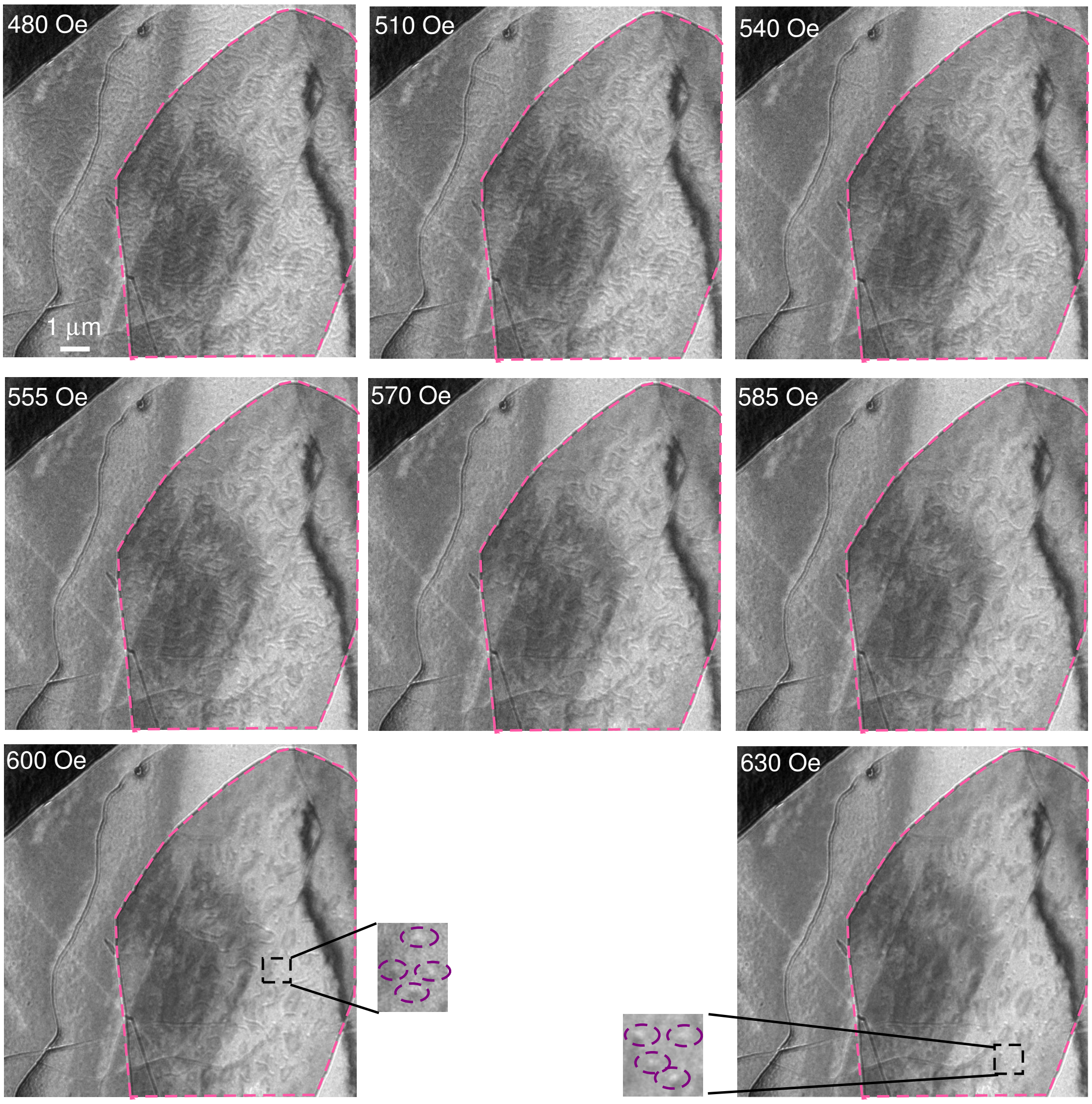

Supplementary Figure 11: Magnetic domain difference for 35L Fe_3GeTe_2 with and without 8L WTe_2 at 195 K with a tilting angle $\alpha = 30^o$ and the detailed field dependence between 480 Oe and 630 Oe.

Supplementary Note 11. Consistency between transport and L-TEM results

At the humps of ρ_{xy}, the total Hall resistivity contains three parts: $\rho_{xy} = \rho_{xy}^{\mathrm{N}} + \rho_{xy}^{\mathrm{AHE}} + \rho_{xy}^{\mathrm{T}}$, where ρ_{xy}^{N} is the normal Hall resistivity, ρ_{xy}^{AHE} is the anomalous Hall resistivity and ρ_{xy}^{T} is the topological Hall resistivity. Assuming a square loop of anomalous Hall effect ($\rho_{xy}^{\mathrm{AHE}} = \rho_{xy}^{\mathrm{Saturated}}$)and a linear ρ_{xy}^{N} at the background, we have: $\rho_{xy} - \rho_{xy}^{\mathrm{Saturated}} = \rho_{xy}^{\mathrm{T}} + \rho_{xy}^{\mathrm{N}} = \frac{1}{ne}(B_{\mathrm{eff}} + B)$. Here, the topological Hall effect is attributed to an effective field,

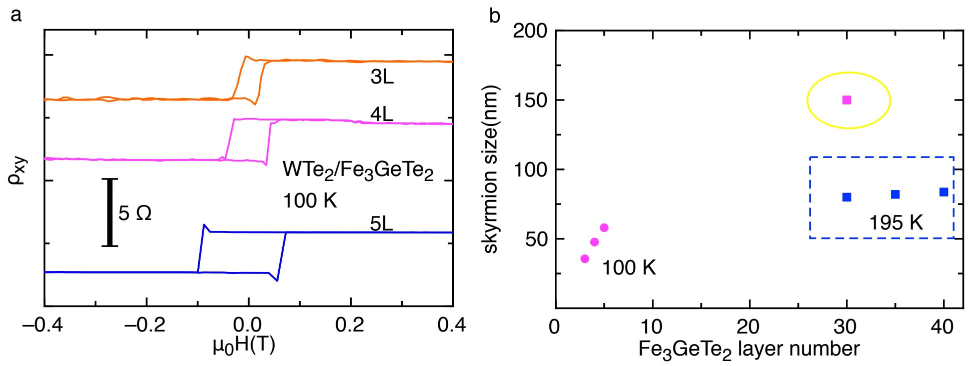

Supplementary Figure 12: Fe_3GeTe_2 thickness dependence of skyrmion size in the WTe_2/Fe_3GeTe_2 heterostructure. a, Topological Hall effect for 1L WTe_2/3L Fe_3GeTe_2, 1L WTe_2/4L Fe_3GeTe_2 and 2L WTe_2/5L Fe_3GeTe_2. b, Extracted skyrmion size from transport and Lorentz transmission electron microscopy as a dependence on Fe_3GeTe_2 thickness at 100 K and 195 K. The points in a circular shape are the skyrmion sizes from topological Hall effect and in a square shape are that from Lorentz transmission electron microscopy. Points in magenta color are taken at 100 K and points in blue color are taken at 195 K.

B_{eff}. Since each magnetic skyrmion contributes a flux quantum, Φ_0, assuming a uniform hexagonal skyrmion lattice, we have $B_{\text{eff}} = \frac{\Phi_0}{\frac{\sqrt{3}}{2}r^2}$, where r represents the skyrmion lattice constant or skyrmion size. The Hall coefficient $\frac{1}{ne}$ can be further obtained from the slope of ρ_{xy} after magnetic saturation. The skyrmion size can therefore be estimated as $r = \sqrt{\frac{\Phi_0}{\frac{\sqrt{3}}{2}\left[\left(\rho_{xy}-\rho_{xy}^{\text{Saturated}}\right)ne-B\right]}}$.

Additional several WTe_2/FGT heterostructures with varied FGT thicknesses show topological Hall loops in Supplementary Fig. 12a. From the topological Hall effect, we obtained the skyrmion lattice constant from these transport signatures at 100 K, as shown by the magenta circles in Supplementary Fig. 12b. It is recognized that such estimation comes from oversimplification of the spin texture and can only provide order-of-magnitude estimation. Fortunately, we have obtained a well-resolved skyrmion lattice in thick (40L) FGT samples at 180 K. The L-TEM observed skyrmion sizes are illustrated by the squares in Supplementary Fig. 12b, where the colors of the squares denote the temperature. Unfortunately, observing domain structures by L-TEM in thin FGT films still fails in our experiment. However, we do see the skyrmion sizes obtained by the two methods fall into the same order of magnitude.

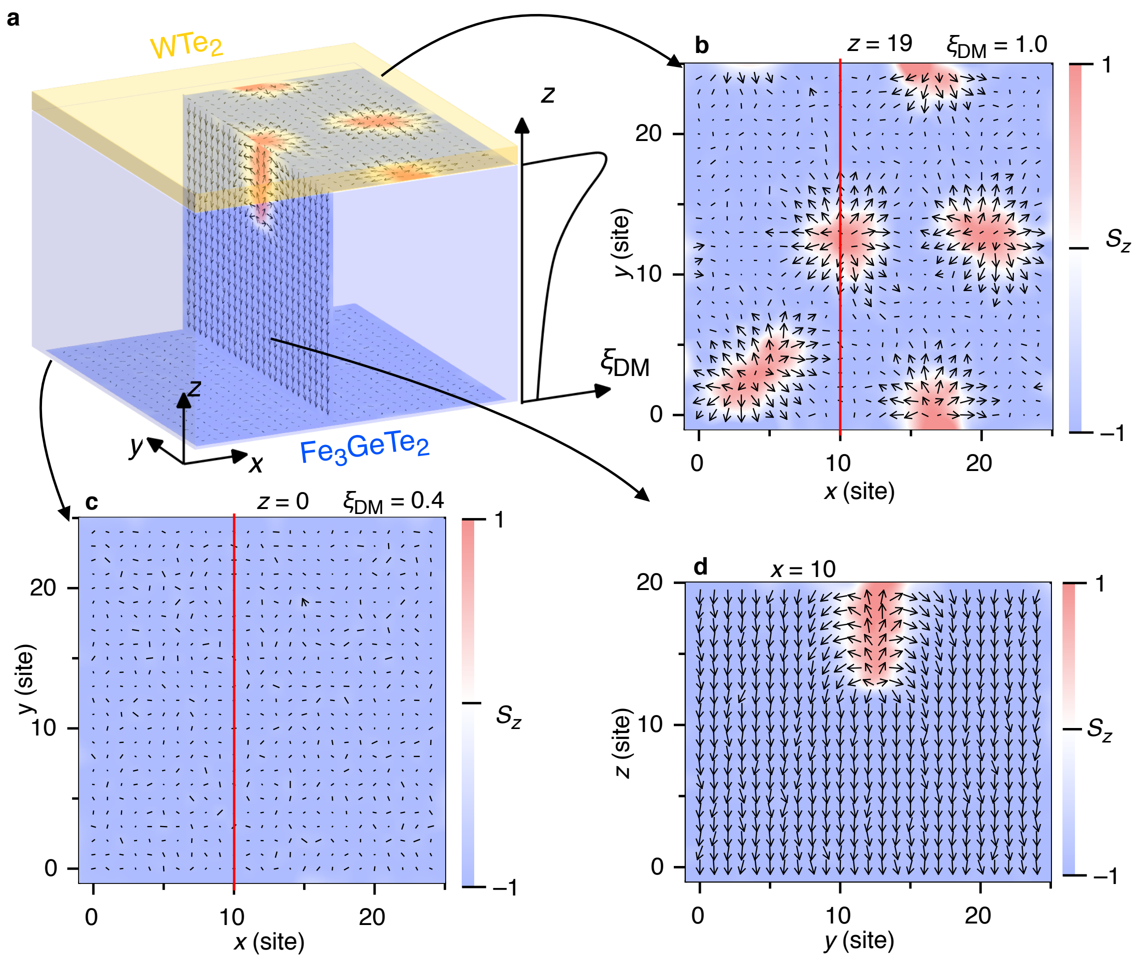

Supplementary Figure 13: 3D view from the simulation of the skyrmions in WTe_2/Fe_3GeTe_2. a, Dzyaloshinskii-Moriya interaction exists at the interface between WTe_2 and Fe_3GeTe_2 and decays when away from the interface. b, Spin polarization at the interface of WTe_2 and Fe_3GeTe_2. c, Spin polarizations for the side of Fe_3GeTe_2 close to SiN substrate. d, Spin polarization at yz plane with a fixed $x = 10$.

On the other side, as shown in Supplementary Fig. 7, the WTe_2 capping can only impact the domain structure for $<$ 65L FGT films, suggesting the presence of a vertical profile of the DMI. Assuming an exponential decay in the DMI profile, our simulation suggests that the skyrmions can only penetrate to a finite depth, where a large volume of ferromagnetic phase shows up away from the interface, as shown in Supplementary Fig. 13. As discussed before, due to frequent scatterings, when carriers pass through the ferromagnetic phase, they quickly lose the memory of the transverse velocity provided by the topological Hall effect,

and therefore the anomalous Hall effect dominates. This explains the missing topological Hall effect in ρ_{xy} humps in thicker films.

Supplementary Note 12. The micromagnetic simulation

The simulation is carried out on a 3D lattice model, with the Hamiltonian written as

$$H = \sum_{\langle i,j \rangle} \left[-J\mathbf{S}_i \cdot \mathbf{S}_j + \mathbf{D}_{i,j}(z) \cdot (\mathbf{S}_i \times \mathbf{S}_j) \right] - \mu_0 \sum_i \mathbf{S}_i \cdot \mathbf{H}_{\mathrm{app}} \tag{3}$$

where J is the Heisenberg exchange coupling, $\mathbf{H}_{\mathrm{app}}$ denotes the applied magnetic field, and the position-resolved Dzyaloshinskii-Moriya interaction $\mathbf{D}_{i,j}(z)$ is given by

$$\mathbf{D}_{i,j}(z) = (\mathbf{z} \times \mathbf{r}_{i,j}) \, D(z), \tag{4}$$

where $D(z) = D_0 \exp\left(\frac{z-t}{l_0}\right)$. Here, t is the thickness of the film and l_0 is a phenomenological penetration depth. The simulation results shown in Fig. 3 in the main text and Supplementary Fig. 13 are carried out on a cubic lattice defined on a $25 \times 25 \times 20$ mesh. The side walls of the mesh are assumed to be periodic boundaries for simplicity. To mimic the case of a thin film, the top and the bottom surfaces are open, that is, to enforce $\mathbf{S}(r) = 0$ for both $z > t$ and $z < 0$. The dynamical behavior of the local spins $\{\mathbf{S}_i\}$ follow the Landau-Lifshitz-Gilbert equation

$$\dot{\mathbf{S}} = -\gamma \mathbf{S} \times \mathbf{H}_{\mathrm{eff}} + \alpha \mathbf{S} \times \dot{\mathbf{S}} \tag{5}$$

where $\gamma = \frac{g}{\hbar}$ is the gyromagnetic ratio and α is the damping factor. The effective field $\mathbf{H}_{\mathrm{eff}}$ is given by $\mathbf{H}_{eff} = -\frac{\partial H}{\partial \mathbf{S}} + \mathbf{L}$, where H is the Hamiltonian given by Eq. 3, and $\mathbf{L}$ is a random field provided by the thermal fluctuation at finite temperature. The dissipation-fluctuation relation $\langle L_\mu(\mathbf{r}, t) \, L_\nu(\mathbf{r}', t') \rangle = \xi \delta_{\mu\nu} \delta_{\mathbf{r}\mathbf{r}'} \delta_{tt'}$ is satisfied, where $\xi = \frac{\alpha k_B T}{\gamma}$, which is determined by the damping factor and the temperature, T, and the average $\langle \cdots \rangle$ is taken over the realizations of the fluctuation field. During the simulation, the applied magnetic field sweeps as a triangle wave, with the slopes much smaller than the characteristic time of the spin dynamics, mimicking an adiabatic scan of the applied field in the experiment. The parameters used in this simulation are $\frac{D_0}{J} = 1$, $k_{\mathrm{B}}T = 0.1J$, and $l_0 = t \ln\left(\frac{D_0}{D_{\mathrm{btm}}}\right)$, where $D_{\mathrm{btm}} = D(z)|_{z=0}$, which is phenomenologically chosen as $D_{\mathrm{btm}} = 0.4D_0$.

References:

---

[1] Deng, Y. *et al.* Gate-tunable room-temperature ferromagnetism in two-dimensional Fe_3GeTe_2. *Nature* **563**, 94 (2018).

[2] Cheng, X. *et al.* Antisymmetric magnetoresistance in magnetic multilayers with perpendicular anisotropy. *Physical Review Letters* **94**, 017203 (2005).

[3] Bodenberger, R. & Hubert, A. Zur bestimmung der blochwandenergie von einachsigen ferromagneten. *physica Status Solidi (a)* **44**, K7–K11 (1977).

[4] Szmaja, W., Grobelny, J., Cichomski, M., Hirosawa, S. & Shigemoto, Y. Magnetic force microscopy investigation of the domain structure of nanocomposite $Nd_2Fe_{14}B/Fe_3B$ magnets. *Acta Materialia* **59**, 531–536 (2011).

[5] Livingston, J. Magnetic domains in sintered Fe-Nd-B magnets. *Journal of Applied Physics* **57**, 4137–4139 (1985).

[6] Gao, Y., Zhu, J., Weng, Y. & Han, B. Domain structure in Fe-implanted $Nd_2Fe_{14}B$ magnets. *Applied Physics Letters* **74**, 1749–1751 (1999).

[7] Stoner, E. C. & Wohlfarth, E. A mechanism of magnetic hysteresis in heterogeneous alloys. *Philosophical Transactions of the Royal Society of London. Series A, Mathematical and Physical Sciences* **240**, 599–642 (1948).

[8] León-Brito, N., Bauer, E. D., Ronning, F., Thompson, J. D. & Movshovich, R. Magnetic microstructure and magnetic properties of uniaxial itinerant ferromagnet Fe_3GeTe_2. *Journal of Applied Physics* **120**, 083903 (2016).
